# Preterm white matter injury: ultrasound diagnosis and classification

**DOI:** 10.1038/s41390-020-0781-1

**Published:** 2020-03-26

**Authors:** Thais Agut, Ana Alarcon, Fernando Cabañas, Marco Bartocci, Miriam Martinez-Biarge, Sandra Horsch, eurUS.brain group, Thais Agut, Ana Alarcon, Roberta Arena, Marco Bartocci, Mayka Bravo, Fernando Cabañas, Nuria Carreras, Olivier Claris, Jeroen Dudink, Monica Fumagalli, Paul Govaert, Sandra Horsch, Alessandro Parodi, Adelina Pellicer, Luca Ramenghi, Charles C. Roehr, Sylke Steggerda, Eva Valverde

**Affiliations:** 1 Department of Neonatology, Hospital Sant Joan de Déu, Institut de Recerca Sant Joan de Déu, Barcelona, Spain; 2 Department of Neonatology, Quironsalud Madrid University Hospital and Biomedical Research Foundation, La Paz University Hospital Madrid, Madrid, Spain; 3 Department of Women’s and Children’s Health, Karolinska University Hospital, Karolinska Institute, Stockholm, Sweden; 4 Department of Paediatrics, Imperial College London, London, UK; 5 Department of Neonatology, Helios Klinikum Berlin Buch, Berlin, Germany; 6 Department Clinical Science Intervention and Technology (CLINTEC), Karolinska Institutet, Stockholm, Sweden; 7 Catholic University of the Sacred Heart, A. Gemelli Hospital, Rome, Italy; 8 Department of Neonatology, La Paz University Hospital, Madrid, Spain; 9 Service de néonatologie et de réanimation néonatale, Hospices Civils de Lyon, Université Claude Bernard Lyon, Villeurbanne, France; 10 UMCU-Wilhelmina Children’s Hospital, Lundlaan 6, 3584 EA Utrecht, The Netherlands; 11 Department of Clinical Sciences and Community Health, University of Milan, Milan, Italy; 12 Fondazione IRCCS Ca’ Granda Ospedale Maggiore Policlinico NICU, Milan, Italy; 13 Department of Neonatology, Erasmus Medical Center University, Sophia Children’s Hospital, Rotterdam, The Netherlands; 14 Department of Neonatology, ZNA Middelheim, Antwerp, Belgium; 15 Department of Rehabilitation and Physical Therapy, Gent University Hospital, Gent, Belgium; 16 Neonatal Intensive Care Unit, Istituto Giannina Gaslini, Via Gaslini 5, 16148 Genoa, Italy; 17 Department of Paediatrics, Medical Sciences Division, Newborn Services, University of Oxford, Oxford, UK; 18 Department of Neonatology, Leiden University Medical Center, Leiden, The Netherlands

## Abstract

White matter injury (WMI) is the most frequent form of preterm brain injury. Cranial ultrasound (CUS) remains the preferred modality for initial and sequential neuroimaging in preterm infants, and is reliable for the diagnosis of cystic periventricular leukomalacia. Although magnetic resonance imaging is superior to CUS in detecting the diffuse and more subtle forms of WMI that prevail in very premature infants surviving nowadays, recent improvement in the quality of neonatal CUS imaging has broadened the spectrum of preterm white matter abnormalities that can be detected with this technique. We propose a structured CUS assessment of WMI of prematurity that seeks to account for both cystic and non-cystic changes, as well as signs of white matter loss and impaired brain growth and maturation, at or near term equivalent age. This novel assessment system aims to improve disease description in both routine clinical practice and clinical research. Whether this systematic assessment will improve prediction of outcome in preterm infants with WMI still needs to be evaluated in prospective studies.

## Introduction

White matter injury (WMI) is the most frequent type of brain lesion in preterm infants and may be present to some degree in up to 50% of very low birth weight infants.^1–4^ Due to improvements in neonatal care, cystic WMI injury, also referred to as cystic periventricular leukomalacia (PVL), has become a rare disease.^5^ On the other hand, the non-cystic, predominantly diffuse form of WMI prevails in very immature infants who survive today.^3–6^ Table 1 presents a glossary with the different terms used to describe WMI of prematurity. Neonatal brain magnetic resonance imaging (MRI) has been shown to be more sensitive than cranial ultrasound (CUS) for the detection of non-cystic WMI, and is therefore considered the gold-standard neuroimaging method to identify and quantify diffuse WMI of prematurity.^7–9^ However, MRI is expensive, it requires transport and in some instances sedation. Scanning is thus challenging, especially in critically ill infants. Furthermore, access to MRI is often limited, which makes serial scanning difficult, and performing a single MRI at term equivalent age in preterm infants may underestimate the severity of WMI.^10^

**Table 1:** Glossary of white matter injury of prematurity.

	Term	Description
Neuropathological abnormalities	Periventricular leukomalacia	Localized necrosis in periventricular (deep) white matter, with loss of all cellular elements. It can evolve over several weeks to cystic lesions (cystic WMI). Much more commonly, focal necrosis is microscopic in size and evolves to glial scars (non-cystic WMI). Both coexist with diffuse gliosis
	Diffuse white matter gliosis	More diffuse preterm white matter changes characterized by a disturbance of early differentiating preoligodendrocytes accompanied by astrogliosis and microgliosis. Oligodendroglial progenitors have disturbed maturation, which interferes with myelination and leads to secondary axonal and cortical injury
Neuroradiological abnormalities	Cystic white matter injury or cystic PVL	Preterm white matter injury characterized by apparent cystic change, the histopathological substrate of which is focal macroscopic necrosis with cystic evolution. CUS is very sensitive to its detection
	PWMLs	MRI finding consisting of localized areas of increased signal intensity on T1-weighted images or decreased signal intensity on T2-weighted images. PWMLs are suggested by inhomogeneous echogenicity seen on CUS. The presence of PWMLs on MRI is sometimes also referred to as “focal non-cystic PVL”
	DEHSI	Qualitative MRI finding seen frequently in preterm infants at term equivalent age consisting of excessive high signal intensity on T2-weighted images. This is associated with increased apparent diffusion coefficient and decreased anisotropy on diffusion-weighted MRI. It is postulated that infants with diffuse white matter injury represent a considerable but unknown proportion of the large group of preterms with white matter loss at term
Additional neonatal cranial ultrasonography terms	Periventricular blush	Bilateral symmetrical relatively hyperechoic areas in white matter typically seen in early scans; most likely representing a normal maturational feature. They include a blush around or below the frontal horn and posterior frontal to parietal blush superior and lateral to the lateral ventricle (the latter referred to as “trigonal blush”). The blush areas are postulated to represent cross-road areas with packed white matter fibers and their accompanying vasculature and/or areas of accumulation of microglial cells. Parallel fibers that are nearly perpendicular to the longitudinal axis of a sonographic beam passing through the anterior fontanel provide multiple interfaces
	Periventricular white matter flaring, flares, echodensities, or hyperechogenicities	Areas of increased echogenicity (as bright or brighter than choroid plexus) in periventricular white matter. These areas can either break down into cysts or resolve after a variable length of time. Transient flares that resolve within the first 7 days of life (or 7 days after the time of the insult) are considered normal, often presumed due to resolving congestion. Underlying neuropathology of pathological flaring is not well known. They could represent inflammatory, ischemic, or hemorrhagic changes in the early stages of white matter injury of prematurity. Persistent densities may represent gliosis.

*PWML* punctate white matter lesions, *DEHSI* diffuse excessive high signal intensity.

CUS has the advantage of being a bedside tool that allows safe, reliable serial imaging. It enables assessment of the evolution of injury over time, as well as brain growth and maturation.^11–13^ Therefore, CUS is the preferred modality for initial and sequential studies in preterm infants. CUS remains very useful for the detection of cystic WMI. Although MRI is superior to CUS for assessing more subtle WMI, in the past decades the quality of neonatal CUS has improved dramatically in terms of resolution and image processing speed. This has broadened the spectrum of preterm white matter abnormalities that can be detected by careful scanning with state-of-the-art CUS systems today. Several evaluation scores have been developed to classify the severity of preterm WMI on MRI.^7,10,14,15^ In contrast, for CUS the only widely used classification system describing the spectrum of WMI/PVL is the one published by de Vries et al. in 1992.^16^ As discussed above, cystic PLV is now uncommon and new high-resolution US machines allow to detect more subtle white matter changes on CUS. The aim of this article is to describe the spectrum of preterm white matter abnormalities that can be detected with modern-day CUS and to propose a novel structured CUS assessment of WMI that seeks to expand the existing classification, accounting for all CUS abnormalities associated with preterm WMI. This novel assessment system aims to improve disease description in both daily clinical routine and clinical studies.

## Pathophysiology of preterm WMI

**Fig. 1:**
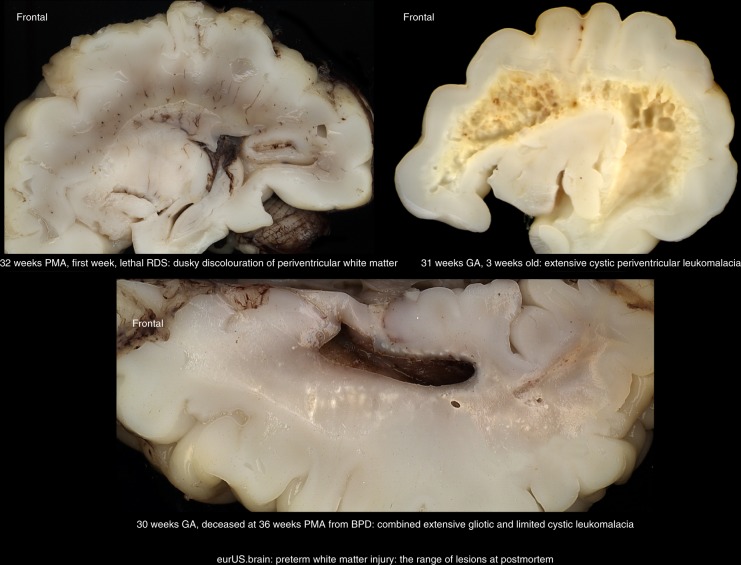
Preterm white matter injury: the range of lesions at postmortem.

The incidence of preterm WMI varies among reports, partly due to the use of different imaging techniques (CUS or MRI) and their particular timelines and diagnostic roles. A recent systematic review showed that prevalence of preterm WMI, including both cystic and non-cystic, was 14.7% based on ultrasound diagnosis and 32.8% based on MRI. The prevalence was 39.6% in infants born below 28 weeks of gestational age, 27.4% below 32 weeks and 7.3% below 37 weeks.^17^ The pathophysiology of WMI of prematurity has been extensively reviewed elsewhere.^18–20^ WMI is related to a confluence of maturational factors that render preterm white matter susceptible to injury.^18^ Known clinical associations are birth asphyxia, hypotension, and both intrauterine and postnatal infection as well as necrotizing enterocolitis.^21–23^ Three overlapping neuropathological variants of preterm WMI have been recognized: cystic WMI with macroscopic focal necrosis evolving to cysts; non-cystic WMI with multiple focal areas of necrosis that evolve into glial scars; and diffuse astrogliosis without focal necrosis (Fig. 1).^1,18,19,24^ To some extent, these variants correlate with distinct clinical outcomes. While classic cystic WMI is typically associated with spastic bilateral cerebral palsy (CP), the predominant deficits following diffuse cerebral WMI are cognitive impairment and behavioral, attention or socialization problems.

At a cellular level, cystic WMI is characterized by focal necrosis in the deep white matter with loss of all cellular elements; in contrast, diffuse non-cystic injury in the central white matter principally affects the pre-oligodendrocyte (pre-OL).^1^ This disturbance consists of pre-OL death or failure of differentiation and, as a result, hypomyelination.^25^ Axonal maturation studies suggest that immature axons are also susceptible to damage.^26,27^ Axonal disease frequently accompanies preterm WMI and is characterized by degeneration of axons and neurons in the brainstem, basal ganglia, thalamus, cerebral cortex and/or cerebellum.^28^ The term ‘encephalopathy of prematurity’ has been proposed to describe the constellation of WMI and the secondary trophic gray matter damage that leads to disturbances in cortical and thalamic development.^29,30^ Reduced volumes of the cerebral cortex and the deep gray nuclei, together with delayed cortical folding and impaired myelination are frequent findings at term equivalent age in preterm infants with WMI.^31–34^

## Ultrasound imaging of preterm WMI

Generally, the presenting feature of preterm WMI on CUS is increased periventricular echogenicity (often named flaring). These periventricular hyperechoic changes can disappear within days or persist longer. When they disappear, they can do so without leaving any abnormality or they can evolve into gliotic or cystic changes and/or ventriculomegaly with other features of brain volume loss and dysmaturation. However, there is still lack of objectivity and difficulties in the interpretation of the changes seen with CUS in the white matter of preterm infants, and sequential CUS appearances of WMI, including cystic but especially non-cystic forms, need further investigation.

### Ultrasound assessment of periventricular hyperechogenicities and cystic WMI

**Fig. 2:**
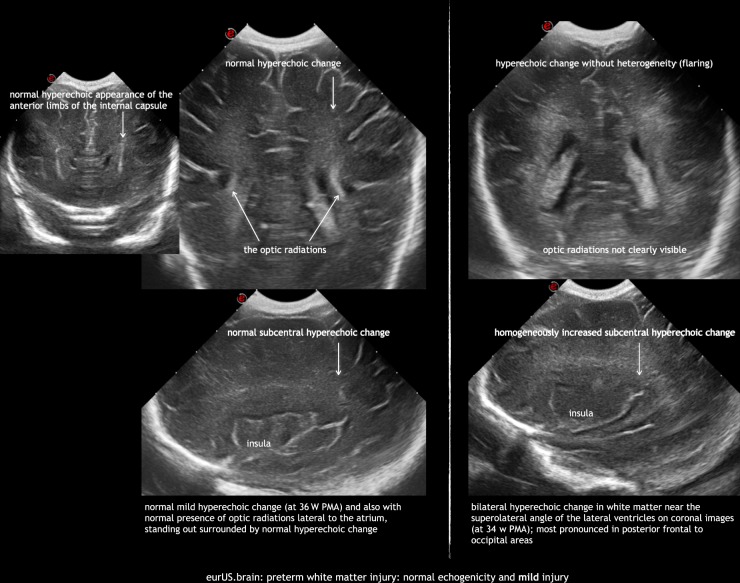
Preterm white matter injury: normal echogenicity and mild injury.

In the early 1980s it became possible to diagnose cystic WMI in preterm infants using CUS.^35^ De Vries et al. described a grading system for PVL that has been widely used:^16^ (I) transient periventricular densities (>7 days); (II) localized cysts besides the external angle of the lateral ventricle; (III) extensive cysts in fronto-parietal and occipital periventricular white matter (cystic PVL); (IV) extensive cysts in subcortical white matter (cystic subcortical leukomalacia). There is evidence of the correlation between this system and prognosis.^36–38^ However, several limitations are encountered when using the classification in clinical practice. Assessment of mild (grade I) WMI remains difficult because periventricular hyperechogenicity is a subjective finding. The interobserver agreement when interpreting hyperechogenicities has been remarkably low in some studies.^39,40^ As de Vries et al. point out, care should be taken not to overdiagnose transient hyperechogenicity.^16^ In general, echogenicity is considered pathological when it is equal to or greater than choroid plexus echogenicity. However, in extremely immature babies the use of this reference is questionable due to the more prominent and echoic choroid plexus in this population and also the fact that cystic PVL can be observed following flaring that never exceeded the brightness of the plexus (Fig. 2). CUS findings as regards periventricular hyperechogenicity that support grade I PVL include patchy appearance, extension of echogenicity beyond the peritrigonal area, and bilateral but asymmetric periventricular distribution.^41^ Homogeneous symmetrical hyperechogenicities are normal around the anterior frontal horns and the parieto-occipital junction of the lateral ventricles, representing the anterior limb of the internal capsule and the optic radiation, respectively.^42,43^

Transient hyperechogenicities disappear within a week without cyst formation or ventricular dilatation. Dammann and Leviton^44^ proposed a classification of hyperechogenicities into brief (1–6 days), intermediate (7–13 days), or prolonged (14 days or more). The duration of periventricular hyperechogenicities correlates with the severity of injury and with long-term outcome, even when they do not evolve into cysts.^45–47^ Prolonged hyperechogenicities have also been found to predict white matter abnormalities on MRI.^8^ However, data regarding prognostic significance of persisting hyperechogenicities are conflicting.^48,49^ Therefore, the use of the term “grade I PLV” in infants with this finding can be problematic, as it may falsely suggest an association with severe motor and cognitive impairment. We recommend instead the more descriptive term “persistent hyperechogenicities” as an image representing mild WMI. It is crucial to look for pathological flaring as described above, note the duration of hyperechoic changes and monitor for subsequent signs of cerebral white matter volume loss. Heterogeneity of white matter echogenicities is important to assess. Inhomogeneous or patchy hyperechogenicities are likely to represent non-cystic WMI and commonly correlate with MRI abnormalities.^50^ Distinction between hyperechogenicities of ischemic or congestive-hemorrhagic nature and their gradual transition into gliosis may be difficult with CUS, but with the use of high-resolution ultrasound probes and wider angles of insonation, it is possible to detect more subtle white matter abnormalities in many cases. Linear hyperechoic changes perpendicular to the ventricle margin often follow deep venous anatomy and have a hemorrhagic component, while globular and coalescing nodules under the central groove correlate more with gliotic changes.

**Fig. 3:**
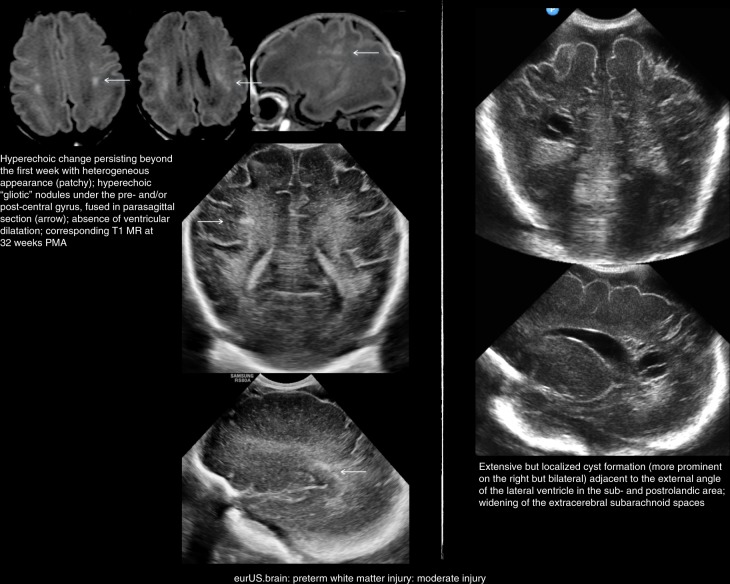
Preterm white matter injury: moderate injury.

**Fig. 4:**
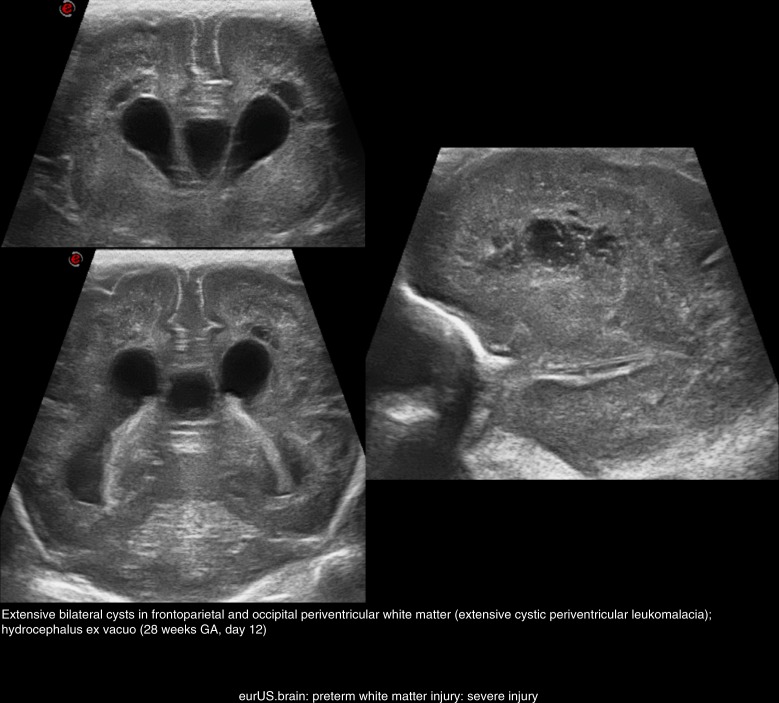
Preterm white matter injury: severe injury.

**Fig. 5:**
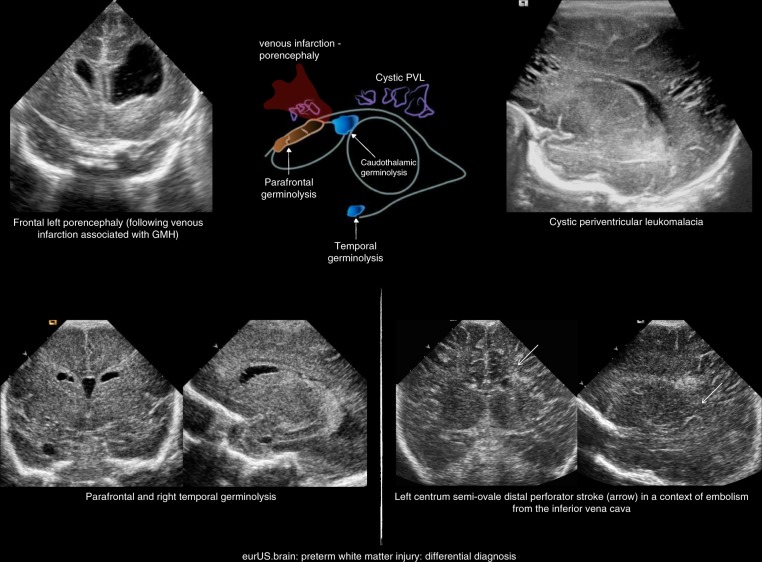
Preterm white matter injury: differential diagnosis.

Serial CUS scans are needed to depict the full natural history of WMI. Examples of mild, moderate, and severe WMI are shown in Figs. 2–4. Cysts typically take 2 to 6 weeks to appear, although timing of cystic changes depends on the severity of WMI.^36,37^ Circumscribed cysts (grade II PVL) should not be confused with frontal pseudocysts due to germinolysis, porencephalic cavitation secondary to venous infarction associated with germinal matrix-intraventricular hemorrhage (GMH-IVH) or arterial infarction involving a terminal branch of a perforator artery (Fig. 5). The extent and especially the location of the cysts are important for predicting outcome: cystic WMI located around the central grove is likely to be associated with spastic bilateral CP, while frontal cysts typically do not associate with CP.^36,51,52^ Disappearance of cysts appears to be due to reabsorption of the fluid within by surrounding brain tissue with subsequent gliosis.^53^ If serial CUS scans are not performed, small cyst may be missed.^10,12,36,37,54^ Ex vacuo ventriculomegaly similar to that seen in diffuse, non-cystic WMI (see below) may be seen instead of cysts in later scans close to term age. A recent large study of extremely preterm infants <27 weeks of gestation showed that in one of six infants with cystic PVL, cysts seen on early CUS (within the first 28 postnatal days) were no longer visible on a later scan, obtained at a mean postmenstrual age of 35 weeks.^54^ When cysts are widespread and extend across the frontal-parieto-occipital regions, they are referred to as grade III PVL. Grade IV or subcortical PVL is a rare condition nowadays. It is also called multicystic encephalomalacia, which often affects the (near) term infants and entails a poor prognosis.

### Ultrasound assessment of diffuse, non-cystic preterm WMI

**Fig. 6:**
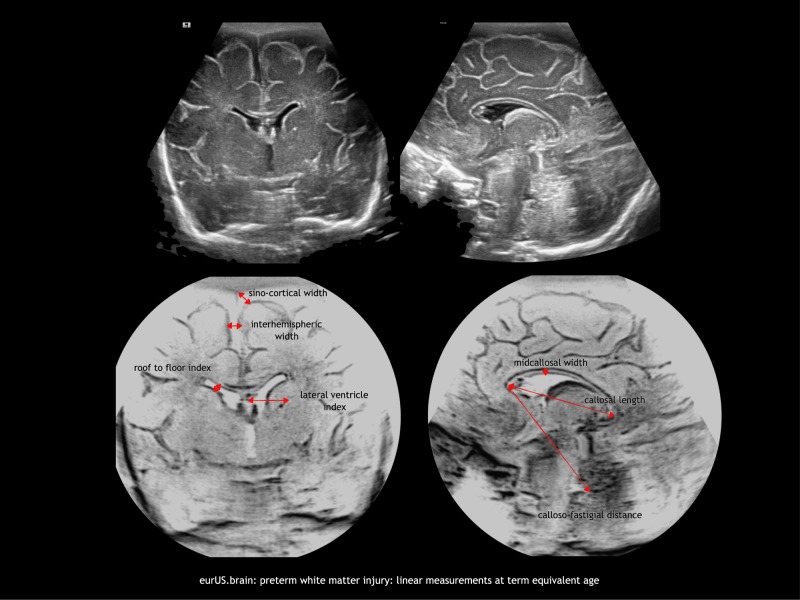
Preterm white matter injury: linear measurements at term equivalent age.

As previously stated, cystic WMI (grade II–IV PVL) is no longer a common finding, and non-cystic WMI has become the dominant form of white matter abnormality in preterm infants. CUS has been shown to have poor sensitivity in the detection of diffuse, non-cystic WMI.^2,3,8,9,55^ However, the quality of CUS imaging has improved remarkably in the past 10–15 years and careful CUS imaging allows to detect a range of white matter lesions. A number of studies established an association between signs of brain atrophy on sequential CUS and poor head growth, reduced brain volumes on MRI, and adverse outcome.^46,49,56–60^ WMI often leads to discrete ventriculomegaly over time. Ventricles develop an irregular shape, without signs of accumulation of cerebrospinal fluid under pressure (i.e. no ballooning). Mild ex vacuo ventriculomegaly with irregular borders, widening of the interhemispheric fissure, and enlarged extracerebral spaces are often seen in late scans of infants with impaired cephalic growth, suggestive of atrophy.^13,49,56^ Sulci may nearly reach the lateral ventricle, and this is accompanied by a reduced complexity of post-primary gyration. These features, which are not included in the de Vries classification system, reflect diffuse WMI and are only present on late scans (near or at term equivalent age). The importance of measurements in late CUS at around term for the prediction of outcome was emphasized in recent studies (refs. ^13,61,62^ and Fig. 6).

### Practical aspects of studying preterm WMI through CUS

Technical aspects must be taken into account in order to increase sensitivity of CUS for the investigation of WMI. Assessment of white matter has improved with the use of high-resolution CUS probes and wider views of insonation. The best way to detect white matter lesions is with a wide sector angle (≥90°) and high-frequency transducer (≥7.5 MHz, preferably linear ≥9 MHz). Appropriate time gain compensation should be set so that subcortical white matter appears isoechoic throughout, both near and distant to the fontanel. Coronal images from the anterior fontanel are better than parasagittal for studying WMI, because the most vulnerable area (which is lateral and superior to the lateral ventricles at the fronto-parietal transition) is more easily seen on coronal views. Tangential studies (in coronal and parasagittal views) help evaluate the extension of WMI in cortico-subcortical regions. To depict cysts, serial scans should be performed at least weekly due to their relatively late appearance and changing features over time.^10,12,36,37,54^ Serial ultrasound imaging is especially crucial in preterm infants with antenatal, intrapartum, or postnatal risk factors for WMI. If information from CUS performed beyond the first 2–4 postnatal weeks is not taken into account, the sensitivity of this neuroimaging tool has been proven to be low compared to conventional MRI for predicting CP and other long-term neurological morbidities.^7,9^ In addition to weekly CUS throughout the neonatal period, a CUS scan should be obtained again near term equivalent age, looking for signs of atrophy or suboptimal brain growth,^9,13,49,57,62^ later also to be confirmed with MRI.^63,64^

### New ultrasound technologies and their present and future role in preterm WMI

Diagnosis of permanent WMI by CUS imaging remains subject to interobserver variability. In order to attain objectivity, medical image processing and computer-aided diagnosis are being developed. These technologies include tools that enhance visual interpretation, automatic segmentation of damaged areas and other regions of interest, performance of automatic measurements, and image registration.^65^ Semi-automated US texture analysis methods have been developed to improve early detection of neonatal WMI.^66,67^ Although they increase sensitivity to detect WMI, their use has not yet been implemented in standard clinical practice.

## MRI-based assessment of preterm WMI combined with CUS

Although this paper focuses on CUS, and MRI appearances of white matter lesions will not be discussed in detail, it is important to understand the complementary role of CUS and MRI when evaluating WMI of prematurity. MRI provides high-resolution depiction of all areas of the brain and is the current reference neuroimaging modality to study the neonatal brain. CUS is suitable for sick or very preterm infants. It reliably detects major brain complications of prematurity (GMH-IVH, hemorrhagic parenchymal infarction and cystic WMI), which are known to predict adverse neurodevelopmental outcomes.^8,37^ Yet, MRI provides better appreciation of the nature and extent of white matter abnormalities, including diffuse WMI and punctate white matter lesions,^2,3,11,55,68^ as well as information on myelination (particularly visualization of the posterior limb of the internal capsule, which at term equivalent age is helpful in predicting motor outcome).^69^ Some neonatal units have adopted brain MRI as standard of care for very preterm infants.^7,13,70,71^ This is usually performed before discharge or around term equivalent age. Some centers undertake two MRI scans, the first one a few weeks after birth, usually at around 32 weeks of postmenstrual age. It is aimed at depicting acute lesions, especially punctate white matter lesions, which tend to be less apparent at term equivalent age. The second MRI study is performed at term equivalent age.^10,14,70^ It is important to note that the appearances of WMI change over time, which has been best documented by serial CUS rather than by serial MRI.^12,38,51^ Performing only MRI may underestimate the severity of injury in preterm infants with WMI, particularly in cases of limited cystic PVL. Therefore, in the assessment of WMI of prematurity, it is crucial to combine CUS and MRI. Furthermore, in most preterm infants with normal CUS at term equivalent age, it is uncertain whether MRI will add any clinically relevant information.^72^ The impact on outcome of mild white matter abnormalities seen on MRI at term equivalent age, which are missed on CUS, is still unknown. High negative predictive values in the absence of major CUS abnormalities for CP at 2 years of age have been reported.^37,73^ Furthermore, when CUS at term equivalent age is assessed systematically, it can reach predictive values for CP and severe cognitive impairment that are in the range of those of term age MRI.^13^ Mild white matter abnormalities seen on MRI at term age are unlikely to change clinical decision making or parental guidance.^61,62^ Indeed, a negative impact on parents of information about such findings has been reported.^74^

A number of MRI evaluation scales have been developed to categorize the severity of preterm WMI Table 2.^7,10,14,15^ Two of these MRI scoring systems are based on findings at around term age, and do not take into account earlier CUS or MRI findings.^7,15^ This allows little comparison between CUS and MRI-based description and categorization of white matter abnormalities. Notably, in the study by Woodward et al.,^7^ white matter cysts were very rare and ventriculomegaly due to post-hemorrhagic ventricular dilatation following a large IVH, or cystic evolution following parenchymal hemorrhage (porencephaly), were part of the WMI scoring system. The classification by Miller et al.^14^ is mainly based on focal signal intensity changes on T1-weighted imaging, related to the number of punctate high signal intensity lesions or regions of low signal intensity suggestive of cysts. The more recent classification published by Martinez-Biarge et al.^10^ takes into account timing of the MRI in relation to postnatal age (or the age after the time of the insult), which allows comparison with categorization based on sequential CUS.

**Table 2:** MRI evaluation scales of the severity of preterm WMI.

	Miller et al.^14^	Woodward et al.^7^	Kidokoro et al.^15^	Martinez-Biarge et al.^10^
Study	Prospective	Prospective	Prospective	Retrospective
Population (*n*) Gestational age (mean) BW (g)	32 VLWB 29 weeks	167 VLBW. Two cohorts. 27.3 weeks; BW 1014 27.1weeks; BW 948	Two cohorts 97 preterm (<30 weeks) (26.7 weeks; BW 949) 22 term healthy (39.1 week; BW 3285)	82 (62 with 2 MRI studies) (29.8 weeks; BW 1453)
Period	2000–2002	Two cohorts: 1998–2000 New Zealand; 2001–2002 Melbourne	2007–2010	2003–2014
Age MRI (PMA = postmenstrual age in weeks)	Two sequential studies Early (31.9 weeks PMA) At term (36.5 weeks PMA)	Term equivalent age	36–42 weeks PMA	138 sequential studies Early (0–2 weeks) Intermediate (2–6 weeks) TEA^a^ (<16 weeks)
MRI acquisition	1.5 T (T1 SE, T2 SE)	1.5 T (T1, T2)	3 T (T1, FSE T2)	1.5 T (T1 IR, T2 DWI)
WM evaluation	Number and size of foci of hyperintensity on T1	Five variables (scores 1–3) 1. WM abnormality 2. Periventricular WM loss 3. Cystic abnormality 4. Ventricular dilatation 5. Thinning of corpus callosum	Six variables (0–4) 1. Cystic lesions 2. Focal abnormality 3. Myelination delay 4. Thinning of corpus callosum 5. Dilated ventricles 6. Volume reduction	Variables depending on MRI 0–2 weeks after birth: focal lesions (T1/DWI) 2–6 weeks after birth: focal or cystic lesions TEA^a^: focal (T1) or cystic, myelination, volume reduction
WMI global score	1. Normal 2. Minimal: 3 or fewer foci <2 mm 3. Moderate: 3 or more foci or area >2 mm, but <5% of the hemisphere 4. Severe: >5% of the hemisphere	1. Normal (scores 5 and 6) 2. Mild (scores 7–9) 3. Moderate (scores 10–12) 4. Severe (scores 13–15)	1. No lesion (scores 0 and 2) 2. Mild (scores 3 and 4) 3. Moderate (scores 5 and 6) 4. Severe (score ≥7) (a score for gray matter and a global brain score are added)	Four grades of WMI taking into account the moment of evaluation
Volume loss evaluation	No	Yes	Yes	Yes (TEA^a^)
Evaluation	Two blinded pediatric neuroradiologist	Two blinded investigators (neuroradiologist, neonatologist)	Single neonatal neurologist (inter- and intraobserver reliability in 20 studies >90%)	NR
Cortical gray matter evaluation	No	1. Signal abnormality 2. Quality of gyral maturation 3. Size of subarachnoid space	1. Signal abnormality 2. Delayed gyration 3. Dilated extracerebral CSF space	No
Deep gray matter evaluation	No	No	Size and signal intensity of basal ganglia and thalami in an axial section	No
Cerebellum evaluation	No	No	Transcerebellar diameter and cerebellar signal intensity	No
Incidence of WMI	56% with WMI: • 31% mild • 21.8% moderate • 3% severe	72% with WMI • 51% mild • 17% moderate • 4% severe (49% with gray matter lesions)	24% with WMI (24% with cerebellar injury)	Selected population
Follow-up	No (comparison with CUS findings only)	Yes Moderate and severe WMI predicted adverse otucome	No	No

^a^Term equivalent age.

## Clinical outcomes of preterm WMI diagnosed by neonatal brain ultrasonography

CUS and MRI yield prognostic information in preterm infants with WMI. Review of studies on prognosis after MRI showing abnormal white matter findings in preterm infants is beyond the scope of this paper. Table 3 summarizes the evidence on the predictive ability for CP of PVL diagnosed by sequential CUS and classified according to de Vries et al.^16^ The neurological morbidities associated with preterm WMI diagnosed by CUS are discussed here. The prognostic significance of hyperechoic changes in periventricular white matter generally depends on whether the abnormality resolves promptly or persists for several weeks. While the prognosis of hyperechogenicities lasting <1 week is accepted to be good, more prolonged flaring is associated with adverse long-term outcomes.^45,46^ In a Dutch series of 44 preterm infants with increased periventricular echogenicity, flaring lasted <7 days in 13, between 7 and 14 days in 18, and more than 14 days in 13 infants.^45^ Four of the children with hyperechogenicities persisting for >14 days developed mild CP (three diplegia and one hemiplegia). Motor performance decreased significantly with increasing duration of flaring, especially regarding lower limb function. No differences in cognitive abilities were found between the groups. Other authors have not found a clear association between periventricular hyperchogenicities and outcome.^48,49^

**Table 3:** Predictive values for cerebral palsy at ≥24 months corrected age of preterm white matter injury diagnosed by sequential CUS.

Author, ref.	GA (weeks)	Grade of PVL according to de Vries et al.^16^	*N*	Cerebral palsy (%)	Predictive values, PVL-II and III vs. PVL-I or normal scan			
					Se (%)	Sp (%)	PPV (%)	NPV (%)
Pierrat et al.^36^	≤32	PVL-II	39	76	–	–	–	–
		PVL-III	27	96				
de Vries et al.^37^	≤32	PVL-I	319	4	60	99.5	77	98.5
		PVL-II	20	59				
		PVL-III	29	94				
Leijser et al.^38^	<32	PVL-I	26	9.5	86	76	50	95
		PVL-II	8	42.5				
		PVL-III	6	75				

*GA* gestational age, *Se* sensitivity, *Sp* specificity, *PPV* positive predictive value, *NPV* negative predictive value, *PVL* periventricular leukomalacia.

The presence of extensive white matter cystic lesions remains the most reliable early predictor for CP, which can be detected by CUS.^36,45,46,75–77^ Furthermore, the severity of cystic PVL correlates with outcome (Table 3). Cyst location is relevant regarding long-term outcomes. Bilateral cysts in the fronto-parietal-occipital or the parieto-occipital regions are associated with a particularly high risk of CP, typically spastic diplegia or quadriplegia, associated with intellectual disability and cerebral visual impairment due to involvement of the optic radiation.^51,78–82^ On the other hand, cysts restricted to the frontal or the anterior parietal lobes normally do not associate CP, even if they are large or extensive.^52^

Moderate non-progressive ventriculomegaly with irregular ventricle margins is a late CUS indicator of diffuse preterm WMI. Infants with this and other signs of atrophy present on the CUS at or near term/discharge achieve lower neurodevelopmental scores.^13,49,57–62^ A recent study of 4193 neonates born at <27 weeks of gestational age found that those with non-hemorrhagic ventriculomegaly detected on CUS close to 36 weeks of postmenstrual age had nearly 3-fold higher odds of neurodevelopmental impairment, cognitive deficit, and moderate to severe CP at 18–22 months corrected age compared with infants with normal CUS.^60^ Alteration in white matter structure and myelination in the preterm infant with WMI has a long-term impact on connectivity, affecting information processing and integration.^83^ A range of impairments in higher cognitive functions, including visual-motor integration, attention, and executive functions, such as working memory, have been found in children born preterm, even in the absence of major brain complications of prematurity.^84,85^ Finally, WMI also underpins behavioral and social-emotional problems often seen in children born very preterm.^84,86–88^

## Proposal of a structured CUS assessment of preterm WMI

Nowadays, high-end ultrasound machines provide high-resolution imaging allowing a more detailed visualization of preterm WMI. When assessing preterm WMI with CUS, it has become relevant to look for sequential signs of non-cystic WMI, such as persistent periventricular hyperechogenicity followed by signs of white matter volume loss and/or impaired brain growth. We propose here a comprehensive CUS assessment system that seeks to systematically evaluate both cystic and non-cystic preterm WMI beyond the grading system available to date (Table 4).

**Table 4:** Proposal of a structured CUS assessment of white matter injury.

Mild	Bilateral hyperechoic change in white matter near the superolateral angle of the lateral ventricles on coronal images (flaring); most pronounced in posterior frontal to occipital areas; gradually disappearing over days	
Moderate	a. Hyperechoic change persisting beyond the first week with heterogeneous appearance (patchy); hyperechoic “gliotic” nodules under the pre- and/or post-central gyrus (=pathological flaring)	Atypical variants Clusters of multiple hyperechoic dots in corona radiata in ELBW infants
	b. Homogeneous hyperechoic change followed by one or two of signs of white matter loss	Atypical variants Homogeneous hyperechoic change without cavitation but with secondary hyperechoic change in pulvinar
	c. Localized cyst formation adjacent to the external angle of the lateral ventricle in the subrolandic area	Atypical variants Isolated bilateral anterior frontal cystic periventricular leukomalacia Isolated bilateral postrolandic cystic periventricular leukomalacia
Severe	a. Hyperechoic change persisting beyond the first week with heterogeneous appearance (patchy) associated with more than two signs of white matter loss in serial scans	
	b. Extensive cysts in fronto-parietal and occipital periventricular white matter (cystic periventricular leukomalacia)	
White matter volume assessment at term equivalent age		
	Coronal measurements indicating frontal white matter loss	Levene ventricular index at the foramen of Monro: >13 mm Roof to floor distance of the frontal horn at the foramen of Monro: >3 mm
	Parasagittal measurements indicating peritrigonal and occipital white matter loss	Midbody >10 mm roof to floor distance thalamo-occipital distance >24 mm
	Measurement of enlarged subarachnoid spaces	Coronal width of the interhemispheric fissure, measured at the foramen of Monro: distance between hemispheres >3 mm Sino-cortical width in coronal view at the foramen of Monro >4 mm
	Measurement of thinning of corpus callosum	Thickness of the body of corpus callosum in midsagittal view <1.5 mm

This novel CUS WMI assessment system is based on serial CUS studies from birth to (near) term age. It includes a detailed assessment of the localization, characteristics, and duration of periventricular white matter hyperechogenicity; a description of cystic changes; and term age CUS findings of white matter volume loss and impaired brain growth. We have included measurements of the lateral ventricles, subarachnoidal spaces, and the corpus callosum that have been previously qualitatively described and related to outcome.^10,13,49^ CUS has been proven to offer reproducible measurements of many structures.^89–91^ We postulate that adding a quantitative assessment of indirect signs of white matter loss and impaired brain growth will decrease rater subjectivity and improve prediction of outcome. We consider CUS to be normal if these measurements are in the normal range and no hyperechogenicities or cysts are present. We define mild WMI as transient hyperechoic changes in the white matter. Moderate WMI consist of hyperechogenic changes that persist over time and evolve to either small focal cysts or one or two signs of white matter volume loss present in late CUS. Finally, when hyperechogenic changes evolve to extensive cystic lesions or lead to extensive white matter loss or significantly impaired brain growth, we consider WMI to be severe.

**Fig. 7:**
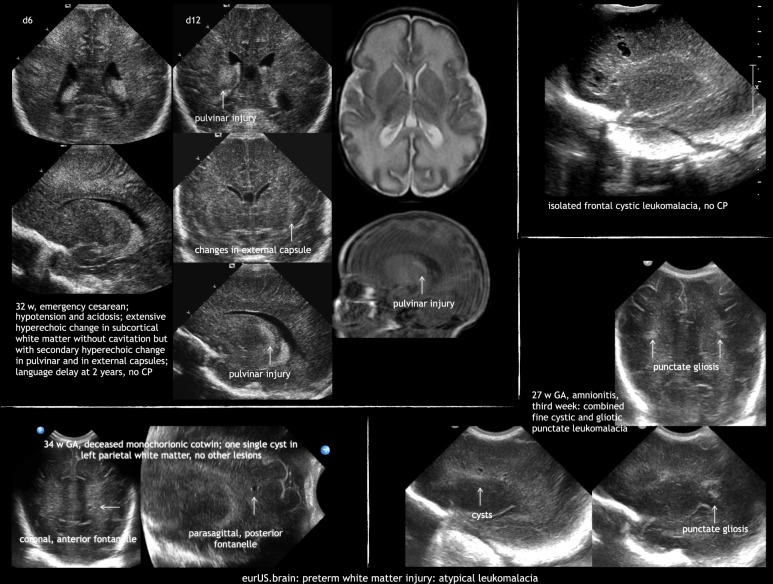
Preterm white matter injury: atypical leukomalacia.

We are aware that some aspects of the proposed classification are not evidence based, but based on expert opinion. Some disease entities are exceptional, but still included in the classification, such as isolated bilateral anterior frontal cystic PVL, isolated bilateral postrolandic PVL, or homogeneous hyperechoic change without cavitation, but with secondary hyperechoic change in the pulvinar (Fig. 7). In our opinion this novel systematic assessment of preterm WMI will improve disease description^92^ and classification in routine clinical practice. Moreover, it will hopefully set the bases to study the full spectrum of preterm WMI assessed with CUS in a more systematic and comprehensive fashion. The ability of the proposed classification to predict long-term neurodevelopmental outcome needs to be established by means of prospective studies.

## Conclusions

Neonatal brain imaging with state-of-the-art CUS devices enables sequential studies and “in vivo” depiction of the natural history of preterm WMI. CUS has been shown to be very sensitive for detecting cystic WMI. However, this form of WMI has become rare in modern neonatology, while the non-cystic form of WMI prevails in very immature infants that survive today. It is therefore important to improve CUS assessment of non-cystic WMI, particularly persistent periventricular hyperechogenicities and indirect sonographic signs of impaired brain development. Such signs of impaired brain growth and maturation take time to evolve, and therefore it is crucial to perform careful serial high-resolution CUS scans from birth to (near) term age. We propose here a novel structured assessment system that seeks to expand the existing classification taking into account the entire spectrum of preterm WMI that can be detected with state-of-the-art CUS devices. This assessment system aims to improve disease description in routine clinical practice and clinical studies. If this novel assessment system will improve prediction of outcome in preterm infants still needs to be evaluated in prospective studies.
